# Comparison between whole mount tissue preparations and virtual tissue microarray samples for measuring Ki-67 and apoptosis indices in human bladder cancer: A cross-sectional study

**DOI:** 10.1097/MD.0000000000004500

**Published:** 2016-08-07

**Authors:** Hisashi Oshiro, Bogdan A. Czerniak, Kentaro Sakamaki, Koji Tsuta, Jolanta Bondaruk, Afsaneh Keyhani, Colin P. Dinney, Takeshi Nagai, Ashish M. Kamat

**Affiliations:** a Department of Pathology, The University of Texas MD Anderson Cancer Center, Houston, TX, USA; b Department of Biostatistics and Epidemiology, Yokohama City University Graduate School of Medicine, Yokohama, Kanagawa, Japan; c Department of Urology, The University of Texas MD Anderson Cancer Center, Houston, TX, USA; d Department of Anatomic Pathology, Tokyo Medical University, Tokyo; e Department of Clinical Sciences and Laboratory Medicine, Kansai Medical University, Hirakata, Osaka; f Department of Pathology, Jichi Medical University, Shimotsuke, Tochigi, Japan.

**Keywords:** Bland–Altman plot analysis, dimensional measurement accuracy, precision, systematic bias, terminal deoxynucleotidyl transferase dUTP nick end labeling, tissue microarray, urinary bladder cancer

## Abstract

Recent tissue microarray (TMA)-based studies have shown that cell proliferation- and apoptosis-related biomarkers are associated with clinical outcomes in patients with bladder urothelial carcinoma. However, little is known about the differences in these biomarker measurements between whole mount tissue preparations and TMAs. This study aimed to elucidate the discrepancy in the measurements of Ki-67 indices (KIs) and apoptosis indices (AIs) between whole mount tissue preparations and TMAs of bladder urothelial carcinoma samples.

Whole mount tissue preparations for Ki-67 immunohistochemistry and terminal deoxynucleotidyl transferase dUTP nick end labeling were made from 30 patients who underwent transurethral resection of bladder urothelial carcinoma. Digital microscopy-assisted virtual TMAs, consisting of 3 small round areas (1 or 0.6 mm in diameter), were generated from the same whole mount tissue preparations. The measurement results in highly reactive areas of biomarkers were compared between the whole mount tissue preparation- and the TMA-based methods. Bland–Altman plot analysis, regression analysis, and Kendall τ were performed to investigate differences in the measurement results, systematic biases, and correlations between biomarkers.

Although the Bland–Altman plot analysis demonstrated that almost all the plots were within the limits of agreement, fixed biases were detected in the 1- and 0.6-mm TMAs for the KI (0.181 and 0.222, respectively) and the AI (0.055 and 0.063, respectively). Proportional biases were also detected in the 1- and 0.6-mm TMAs for the AI (*P* < 0.001 and *P* < 0.001, respectively). Furthermore, positive correlations between KIs and AIs were observed in whole mount tissue preparations (*r* = 0.260, *P* = 0.044) and in the 1 mm TMAs (*r* = 0.375, *P* = 0.004); however, no such correlation was observed in the 0.6 mm TMAs.

Our study suggests that the measurement results for certain biomarkers of bladder urothelial carcinoma obtained from TMA-based samples can be susceptible to systematic bias, and the lack of correlation between biomarkers cannot be avoided as it is in whole mount tissue preparations. Virtual TMAs can help identify systematic bias and establish a better sampling strategy prior to performing high-throughput TMAs for biomarker studies.

## Abstract 2

Supplemental Digital Content is available in the text

## 1 Introduction

Biomarkers may help stratify bladder cancer patients to provide them with appropriate therapeutic strategies. Recent studies have demonstrated that cell proliferation- and apoptosis-associated biomarkers are useful for predicting the clinical outcomes of patients with bladder urothelial carcinoma.^[1]^ The cell proliferation marker Ki-67 is a nuclear antigen expressed in the S, G1, G2, and M phases of the cell cycle. A study of patients who underwent transurethral resection for nonmuscle invasive bladder carcinoma demonstrated that a high Ki-67 index (KI) was an independent risk factor for disease recurrence and progression using a KI cut-off of 25%.^[2]^ Other studies showed that with a cut-off of 20%, a high KI was associated with advanced pathological stage, higher tumor grade, lymphovascular invasion, metastasis, disease recurrence, and stage-adjusted disease-specific mortality in patients with bladder urothelial carcinoma who underwent radical cystectomy.^[3,4]^ A low apoptosis index (AI) was associated with worse local control or a lower survival rate in patients with bladder urothelial carcinoma.^[5–7]^ Similarly, a previous study demonstrated that low expression of cleaved caspase-3, a key apoptosis marker, was associated with a low survival rate in patients with bladder carcinoma.^[8]^

However, some studies do not correspond with these results.^[9–12]^ The use of different cut-off points in various studies has certain clinical implications, making study comparisons difficult.^[13]^ The causes of such discrepancies are thought to be differences in sampling, fixation, antigen retrieval, cell counting, tissue section thickness, antibody concentration, and/or reaction time protocols.^[1,14,15]^ Of these factors, careful attention must be paid to sampling methodology because most biomarker studies of bladder urothelial carcinoma are now based on tissue microarrays (TMAs).^[16]^

TMA studies for bladder urothelial carcinoma typically use 2 or 3 core samples (1 or 0.6 mm in diameter) per case.^[16]^ If the TMA-based measurement results for biomarkers are sufficient for clinical interpretation, then we can replace whole mount tissue preparations with TMA samples or use the 2 methods interchangeably. One approach to explore this issue is a virtual TMA investigation, which is a simulation study that uses virtual cores constructed from images taken from whole mount tissue preparations and does not require the physical punching of actual holes in donor paraffin blocks.^[17,18]^ Although previous biomarker studies using virtual TMAs have compared measurement results obtained using whole mount tissue preparations and TMAs for breast carcinoma^[18]^ and peripheral T-cell lymphoma,^[17]^ no adequate data exist to demonstrate that bladder urothelial carcinoma TMAs produce acceptable measurements compared with conventional whole mount tissue preparations.

The aim of the present study was to elucidate the degree of discrepancy in the KI and AI measurement results acquired from whole mount tissue preparations and virtual TMAs of human bladder urothelial carcinoma when each measurement was obtained using constant measurement criteria.

## 2 Materials and methods

### 2.1 Case selection

This retrospective study was approved by the Institutional Review Board of the University of Texas MD Anderson Cancer Center (LAB07-0420). The required sample size for this study was estimated as 30, which we thought adequate for our study purpose and statistical interpretation based on the literature.^[19–22]^ A total of 30 patients (10 Ta, 10 T1, and 10 T2–T4 patients) were retrospectively and consecutively retrieved from the computerized database of MD Anderson Cancer Center between 1998 and 2008. For inclusion in the study, patients were required to have undergone transurethral resection of bladder urothelial carcinoma for the 1st time at MD Anderson Cancer Center without receiving systemic chemotherapy, immunotherapy, radiation therapy of the pelvic region, or any intravesical therapies, including Bacillus Calmette–Guerin vaccine, prior to initial transurethral resection. The exclusion criterion was unavailability of formalin-fixed, paraffin-embedded tissue samples from the initially resected bladder tumor. Board-certified urologists reviewed patients’ medical charts and confirmed information regarding patient age, gender, clinical history, clinical imaging, and treatment for bladder cancer. Board-certified anatomic pathologists evaluated histological samples and confirmed patients’ pathological diagnoses and tumor grades in accordance with standards in the literature.^[23]^

### 2.2 Histopathological slide preparation

Among the initially resected bladder urothelial carcinoma samples, 1 formalin-fixed, paraffin-embedded tissue block was selected per patient by board-certified anatomic pathologists. Each block was sectioned at a thickness of 4 μm, and sections were mounted onto silane-coated glass slides and used for immunohistochemistry (for KI) and terminal deoxynucleotidyl transferase dUTP nick end labeling (for AI).

Immunohistochemistry was performed on whole mount tissue sections using a Ki-67 antibody (SP6; Lab Vision; Fremont, CA; 1:200 dilution) and an automated immunostainer (Nemesis 8400; Biocare Medical; Concord, CA). Sections and appropriate positive and negative control samples were first subjected to antigen retrieval using citrate buffer (pH 6.0) for 20 minutes at 95 to 100 °C, followed by incubation with primary antibody for 60 minutes at room temperature and colorization using an avidin–biotin complex detection method and diaminobenzidine, with procedures conducted in accordance with the manufacturer's instructions and methods described in the literature.^[24]^

Terminal deoxynucleotidyl transferase dUTP nick end labeling was performed on whole mount tissue sections using an Apoptosis in situ Detection Kit (Wako; Osaka, Japan) with diaminobenzidine colorization; appropriate control samples were used, and procedures were performed in accordance with the manufacturer's instructions and methods described in the literature.^[25,26]^

### 2.3 Production of virtual TMAs

Each whole mount tissue preparation was used to compare different sampling methods for the KI and AI: a whole mount tissue preparation-based method; and a TMA-based method consisting of 3 small round areas (cores) of either 1 or 0.6 mm in diameter that were selected from the same whole mount tissue preparations, which we called “virtual TMAs.”

**Figure 1:**
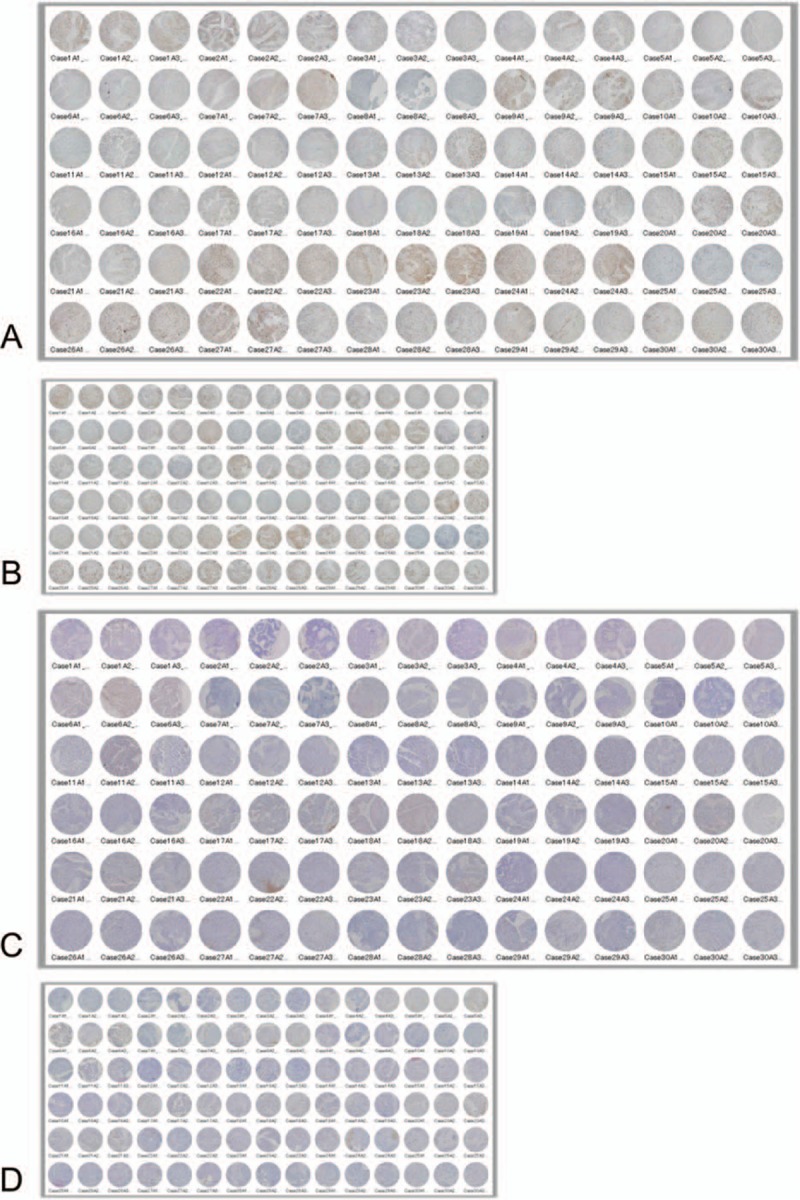
Virtual tissue microarrays used in this study. (A) Ninety core samples with a diameter of 1 mm (Ki-67 immunohistochemistry). (B) Ninety core samples with a diameter of 0.6 mm (Ki-67 immunohistochemistry). (C) Ninety core samples with a diameter of 1 mm (terminal deoxynucleotidyl transferase dUTP nick end labeling (TUNEL). (D) Ninety core samples with a diameter of 0.6 mm (TUNEL).

First, we made a small round grid with Excel (Microsoft Japan; Tokyo, Japan), in which each small, round area was assigned a number. Second, we printed the grid onto a transparent film such that the diameter of each small, round area was 1 or 0.6 mm. Third, we placed the transparent film on each whole mount tissue preparation and divided the bladder cancer tissue into small, round areas. Finally, for each whole mount tissue preparation, we used a random number table to randomly select 3 small, round areas in which at least one third of the area contained neoplastic cells and then took digital photomicrographs. Thus, virtual TMAs were composed of samples with a diameter of 1 or 0.6 mm (Fig. 1 and Supplementary Figure 1).

### 2.4 Histopathological image analysis

All KI and AI measurements were performed twice by a board-certified anatomic pathologist (HO) with more than 15 years of experience. The rater measured the KI and AI of each image in the following order with an interval of at least 2 weeks between the different sample types: whole mount tissue preparations (as a reference standard), 1 mm TMA samples (as an index test), and 0.6 mm TMA samples (as an index test). The rater was blinded to the results of the reference standard and the index tests for the KI and AI until all measurements were completed.

**Figure 2:**
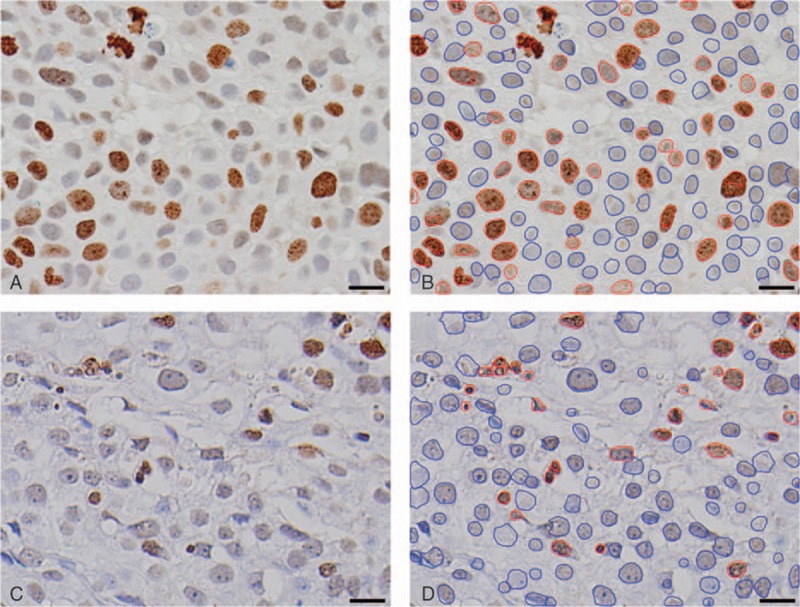
Photomicrographs of bladder cancer tissue used for digital microscope-assisted morphometry. (A) Ki-67 immunohistochemistry (40 × objective; scale bar: 20 μm). (B) Ki-67 immunohistochemistry analyzed by NuclearQuant (3DHISTECH; Budapest, Hungary). Ki-67-negative nuclei are outlined in blue, whereas Ki-67-positive nuclei are outlined in red (40 × objective; scale bar: 20 μm). (C) Terminal deoxynucleotidyl transferase dUTP nick end labeling (TUNEL) (40 × objective; scale bar: 20 μm). (D) TUNEL analyzed by NuclearQuant, TUNEL-negative nuclei are outlined in blue, whereas TUNEL-positive nuclei are outlined in red (40 × objective; scale bar: 20 μm).

We used widely accepted measurement protocols for KI and AI based on the literature.^[3,5,6,27,28]^ Briefly, in the whole mount histological preparations and virtual TMA samples, highly reactive areas, which we called “hot spots,” were observed under a light microscope using an imaging system (DP70; Olympus; Tokyo, Japan). A high-power field (215 μm × 160 μm = 3440 μm^2^) image was digitally obtained, saved in tagged image file format (1020 × 768 pixels), and converted into MRXS files by Slide Converter (3DHISTECH; Budapest, Hungary). These digital images were viewed with the assistance of Pannoramic Viewer (3DHISTECH) and NuclearQuant (3DHISTECH) image analysis software under detailed measurement settings (Fig. 2). After reviewing, adjusting and confirming the computer graphic data, the most highly reactive images were selected to determine the KI and AI. The numbers of positive and total neoplastic cells counted in each image were added in descending order of the ratio of positive cells to total cells until the total number of neoplastic cells exceeded 999. If the 3 microarray samples did not contain more than 999 neoplastic cells, all the neoplastic cells in the 3 microarray samples were counted to determine the KI or AI. For WMTPs, we initially measured numerous potential hot-spot high-power field images (for the KI, an average of 12.5 images per case; for the AI, an average of 14.6 images per case). After measuring and ranking these images, we selected hot-spot images in descending order (for the KI, an average of 7.6 images per case; for the AI, an average of 7.0 images per case). For virtual TMAs, we initially measured many more potential hot-spot images (for the KI, an average of 14.5 images from 1 mm TMAs per case and 13.5 images from 0.6 mm TMAs per case; for the AI, an average of 14.0 images from 1 mm TMAs per case and 12.3 images from 0.6 mm TMAs per case) than the number of hot-spot high-power images that were eventually selected (for the KI, an average of 8.0 images from 1 mm TMAs per case and 7.6 images from 0.6 mm TMAs per case; for the AI, an average of 7.5 images from 1 mm TMAs per case and 7.6 images from 0.6 mm TMAs per case).

### 2.5 Statistical analyses

The 1st and 2nd measurement values were used to assess intrarater reliability, and the mean values were used for the Bland–Altman plot analysis and Kendall τ. For the assessment of intrarater reliability, the intraclass correlation coefficients for a single measurement and for the average of 2 measurements were investigated using IBM SPSS Statistics 21 (IBM Japan; Tokyo, Japan). Bland–Altman plot analysis^[19,29,30]^ was used to compare the 2 measurement methods using MedCalc 14 (MedCalc; Ostend, Belgium). In addition, ordinary least-squares regression analysis was performed on the Bland–Altman plot using MedCalc 14. Fixed bias was indicated if the 95% confidence interval (CI) for the mean value of the difference did not contain 0. Proportional bias was indicated if the slope of the ordinary least regression of the differences in the means differed significantly from 0 (*P* < 0.05). Kendall τ was used to examine correlations between KIs and AIs in the whole mount tissue preparations and in the virtual TMA samples using IBM SPSS Statistics 21. In all statistical analyses, a *P*-value of less than 0.05 (2-sided) was considered to indicate statistical significance.

## 3 Results

A total of 30 patients (10 Ta, 10 T1, and 10 T2-T4 patients) fulfilled our inclusion criteria, and none of the patients were eliminated based on our exclusion criterion. The patients’ characteristics are summarized in Table 1. Our study population had a mean age of 65.9 years (range, 45–89), the men to women ratio was 24:6, and 4 patients had minor histological components (glandular, micropapillary, or squamous differentiation) other than urothelial carcinoma.

**Table 1:**
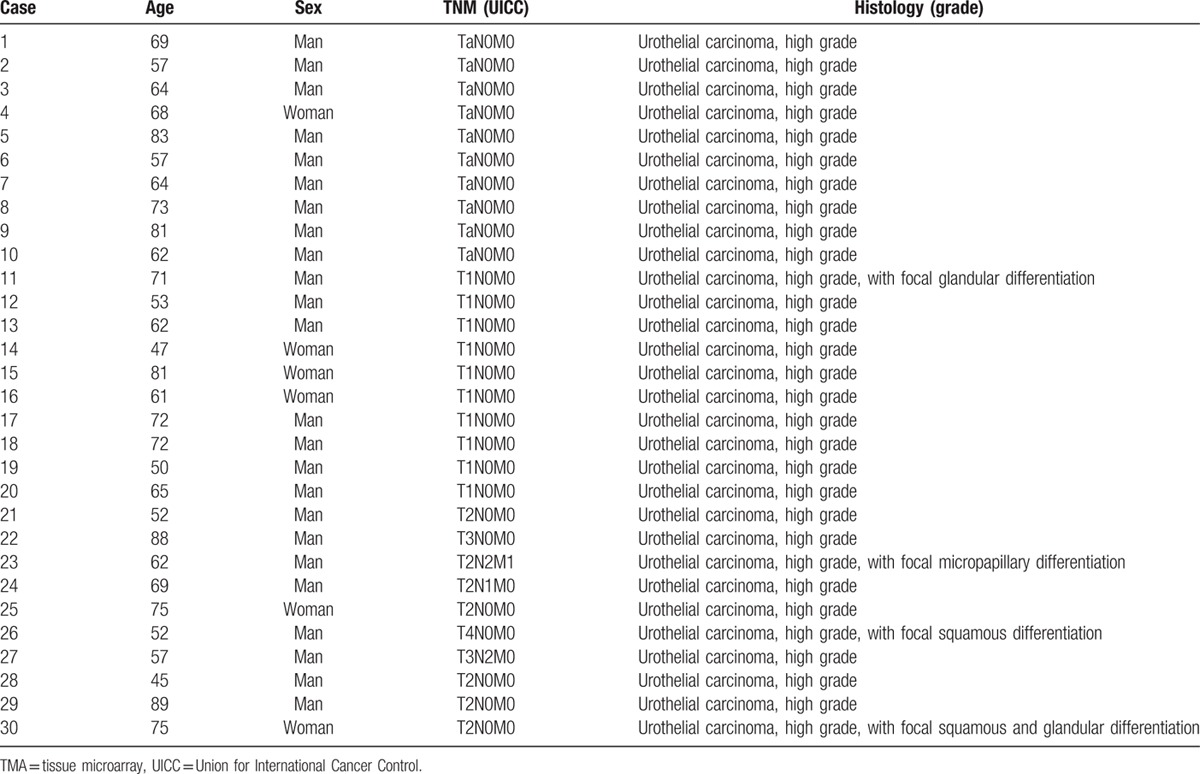
Clinicopathological features of 30 cases with bladder carcinomas.

The 1st and 2nd measurement results are shown in Table 2. The intraclass correlation coefficients for a single KI measurement and for the average of 2 KI measurements were 0.987 (95% CI, 0.972–0.994, *P* < 0.001) and 0.993 (95% CI, 0.986–0.997, *P* < 0.001) in the whole mount tissue preparations, 0.995 (95% CI, 0.989–0.997, *P* < 0.001) and 0.997 (95% CI, 0.994–0.999, *P* < 0.001) in the 1 mm TMA samples, and 0.991 (95% CI, 0.982–0.996, *P* < 0.001) and 0.996 (95% CI, 0.991–0.998, *P* < 0.001) in the 0.6 mm TMA samples, respectively. The intraclass correlation coefficients for a single AI measurement and for the average of 2 AI measurements were 0.989 (95% CI, 0.978–0.995, *P* < 0.001) and 0.995 (95% CI, 0.989–0.997, *P* < 0.001) in the whole mount tissue preparations, 0.992 (95% CI, 0.984–0.996, *P* < 0.001) and 0.996 (95% CI, 0.992–0.998, *P* < 0.001) in the 1 mm TMA samples, and 0.994 (95% CI, 0.989–0.997, *P* < 0.001) and 0.997 (95% CI, 0.994–0.999, *P* < 0.001) in the 0.6 mm TMA samples, respectively.

**Table 2:**
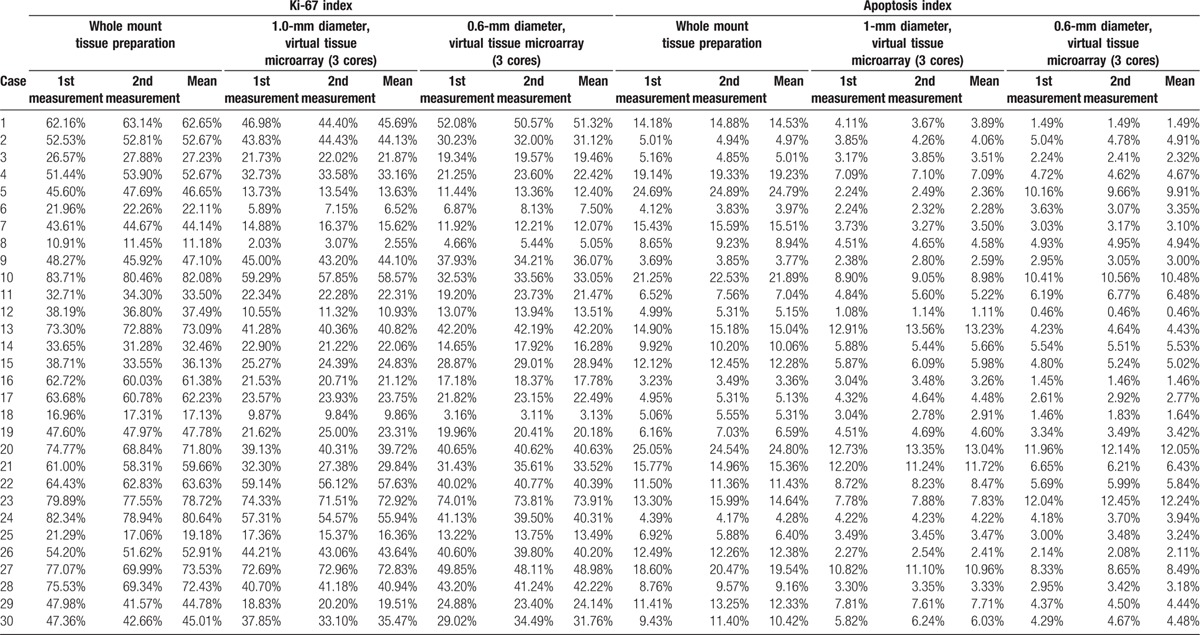
Ki-67 and apoptosis indices in bladder carcinomas.

**Figure 3:**
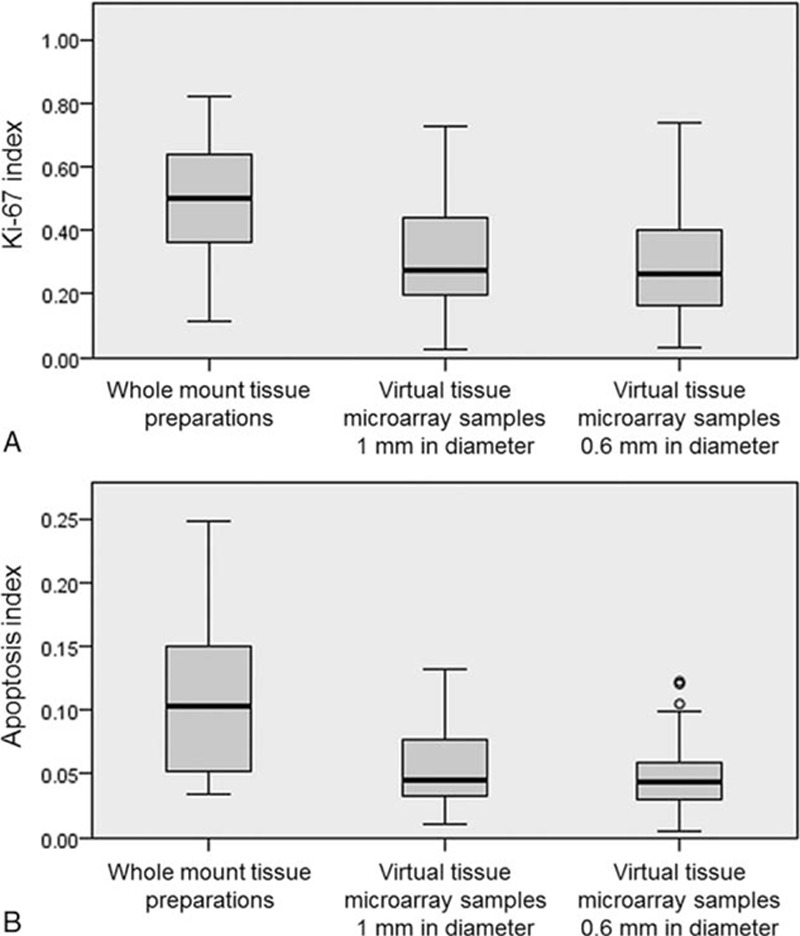
Boxplots of KI and AI. (A) KI measured using WMTPs and virtual TMA samples with diameters of 1 and 0.6 mm. (B) AI measured using WMTPs and virtual TMA samples with diameters of 1 and 0.6 mm. AI = apoptosis index, KI = Ki-67 index, TMA = tissue microarray, WMTP = whole mount tissue preparation.

As shown in Fig. 3, the KIs measured from the whole mount tissue preparations were as follows: median, 0.502; minimum, 0.112; maximum, 0.821; and interquartile range, 0.302. The KIs measured using the 1 mm virtual TMA samples were as follows: median, 0.273; minimum, 0.026; maximum, 0.729; and interquartile range, 0.254. The KIs measured using the 0.6 mm virtual TMA samples were as follows: median, 0.265; minimum, 0.031; maximum, 0.739; and interquartile range, 0.247. Only case number 25 did not contain more than 999 neoplastic cells in the 0.6 mm TMA samples for the KI assessment (1st measurement, 94/711 = 0.132; 2nd measurement, 99/720 = 0.138). The other TMA samples all contained more than 1000 neoplastic cells.

The AIs measured in the whole mount tissue preparations were as follows: median, 0.102; minimum, 0.034; maximum, 0.248; and interquartile range, 0.010. The AIs measured using the 1 mm virtual TMA samples were as follows: median, 0.045; minimum, 0.011; maximum, 0.132; and interquartile range, 0.044. The AIs measured using the 0.6 mm virtual TMA samples were as follows: median, 0.044; minimum, 0.005; maximum, 0.122; and interquartile range, 0.031.

**Figure 4:**
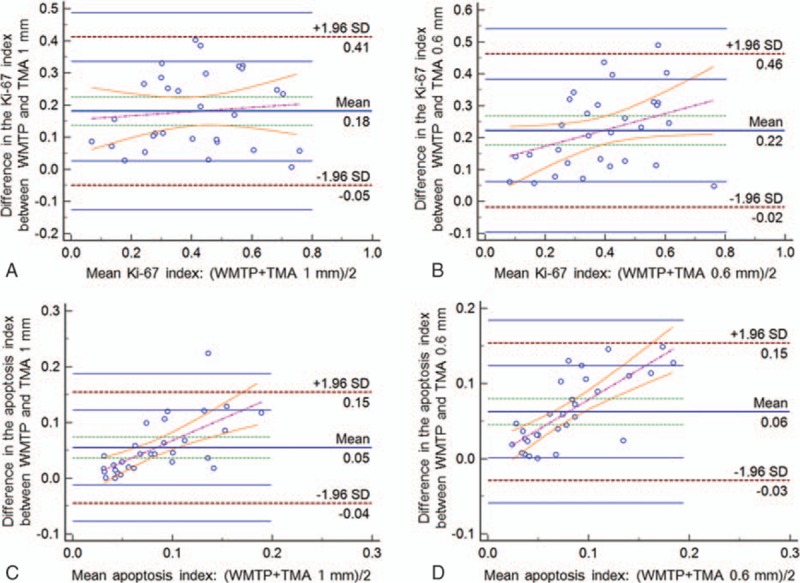
Bland–Altman plots for the comparison of different measurement methods. Each plot shows the differences between the 2 methods against the averages of the 2 methods; the lines represent the mean differences, 95% CI of the mean differences, upper and lower limits of agreement (mean differences ± 1.96 SD), 95% CI of the upper and lower limits of agreement, and regression of the differences. (A) Comparison of the KI measured using WMTPs or virtual TMA samples with a diameter of 1 mm (TMA 1 mm). (B) Comparison of the KI measured using WMTPs or virtual TMA samples with a diameter of 0.6 mm (TMA 0.6 mm). (C) Comparison of the AI measured using WMTPs or virtual TMA samples with a diameter of 1 mm (TMA 1 mm). (D) Comparison of the AI measured using WMTPs or virtual TMA samples with a diameter of 0.6 mm (TMA 0.6 mm). AI = apoptosis index, CI = confidence interval, KI = Ki-67 index, SD = standard deviation, TMA = tissue microarray, WMTP = whole mount tissue preparation.

Figure 4A shows a comparison of the KIs obtained using whole mount tissue preparations and 1 mm TMA samples. Although all the plots were within the limits of agreement, a fixed bias was indicated by the mean difference of 0.181 (95% CI: 0.137–0.225). However, a proportional bias was not evident (slope: 0.065, *P* = 0.594).

Figure 4B shows a comparison of the KIs obtained using whole mount tissue preparations and 0.6 mm TMA samples. Although nearly all the plots were within the limits of agreement, a fixed bias was indicated by the mean difference of 0.222 (95% CI: 0.176–0.268), but a proportional bias was not evident (slope: 0.256, *P* = 0.555).

Figure 4C shows a comparison of the AIs obtained using whole mount tissue preparations and virtual 1 mm TMA samples. In this comparison, although almost all the plots were within the limits of agreement, a fixed bias was indicated by the mean difference of 0.055 (95% CI: 0.036–0.074), and a proportional bias was detected (slope: 0.772, *P* < 0.001).

Figure 4D shows a comparison of the AIs obtained using whole mount tissue preparations and the 0.6 mm TMA samples. Although all the plots were within the limits of agreement, a fixed bias was indicated by the mean difference of 0.063 (95% CI: 0.045–0.080), and a proportional bias was detected (slope: 0.798, *P* < 0.001).

**Figure 5:**
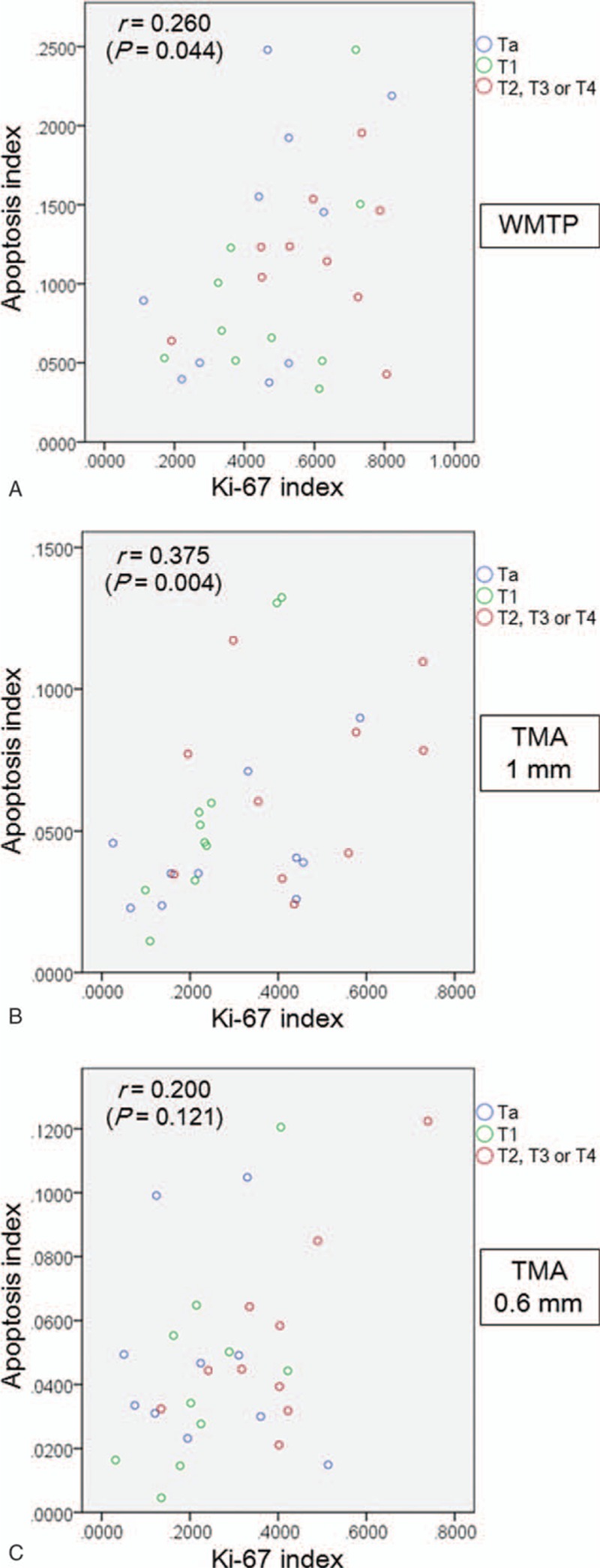
Correlation between KI and AI in WMTPs (A), virtual TMA samples with a diameter of 1 mm (B), and virtual TMA samples with a diameter of 0.6 mm (C). AI = apoptosis index, KI = Ki-67 index, TMA = tissue microarray, WMTP = whole mount tissue preparation.

Scatter plots to investigate the correlation between KIs and AIs are shown in Fig. 5. A positive correlation between KIs and AIs was observed in the whole mount tissue preparations (*r* = 0.260, *P* = 0.044) and the 1 mm virtual TMA samples (*r* = 0.375, *P* = 0.004); however, a correlation was not observed in the 0.6 mm virtual TMA samples (*r* = 0.200, *P* = 0.121).

## 4 Discussion

In the present study, the intraclass correlation coefficients for a single measurement and for an average of 2 measurements for the KI and AI were sufficient to support intrarater reliability according to the proposed criteria.^[31]^ In the Bland–Altman plot analysis, differences between the KI and AI measurements in the TMA samples and whole mount tissue preparations were nearly all within the limits of agreement or the 95% CIs of these limits. However, we found fixed biases in the KI measurements and fixed and proportional biases in the AI measurements. A smaller TMA sample diameter was correlated with a larger systematic bias.^[32]^ Our findings clearly demonstrated that these indices differed when they were measured using virtual TMA samples or whole mount tissue preparations.

There are 2 main types of error that interfere with research inferences: random error and systematic error. Random error is a wrong result due to chance; sources of variation are equally likely to distort estimates in the study in any direction.^[32]^ Among several techniques for reducing the influence of random error, the simplest is to increase the sample size.^[32]^ Systematic error is a wrong result due to bias; sources of variation distort the study findings in 1 direction.^[32]^ Increasing the sample size has no effect on reducing systematic error.^[32]^ Systematic error between different sampling methods can represent a fixed and/or a proportional bias. The only way to improve the accuracy of the estimate is to design the study in such a way that either reduces the size of the various biases or provides some information about them.^[32]^ Although ordinary least-squares regression analysis is used in Bland–Altman plots to estimate the slope, caution is required when evaluating the fixed bias if a proportional bias exists. If a proportional bias exists (slope ≠ 0) and the mean difference almost inevitably deviates from zero, there is a risk that fixed bias will be overestimated.^[30]^ If a proportional bias exists in 1 direction (e.g., slope > 0) and a fixed bias exists in the opposite direction, there is a risk that the fixed bias will be underestimated.^[33]^

In the present study, the mean difference in the KI deviated considerably from zero (18.1% in the 1 mm TMA samples and 22.2% in the 0.6 mm TMA samples). Regarding the AI measurements, we found both fixed and proportional biases, although the fixed bias might have been overestimated due to the presence of a proportional bias. Hence, special attention should be paid to assigning cut-off points for clinical applications when using data obtained from different samplings of TMAs.^[1–12,14,34]^

We observed a positive correlation between KIs and AIs in whole mount tissue preparations and 1 mm virtual TMA samples; to the best of our knowledge, this had not yet been reported for human bladder cancer tissue. However, there was no correlation in the 0.6 mm microarray samples, indicating that smaller samples can result in a failure to detect a potentially important relationship between certain biomarkers. The likely cause of this phenomenon is that smaller samples are influenced by heterogeneous biomarker expression; therefore, much greater numbers of cores from sites containing adequate tumor cells would likely be required to obtain concordant results for WMTPs, 0.6 mm TMAs, and 1 mm TMAs. Because heterogeneity in expression appears to depend on the characteristics of the biomarker itself and the cells that are tested, such a discordance will be minimized when using homogeneously expressed biomarkers in TMA studies. If used carefully in this regard, TMA studies still have merit in that they are a low-cost, high-throughput method for determining the necessity for future detailed investigations between biomarkers and clinical outcomes.^[16]^

There are several limitations to the present study. First, we utilized only 1 counting protocol, as described in the methods section. Factors influencing measurement results by a single rater in this type of biomarker study may include the following: observational field areas depending on the magnification (e.g., 200 × or 400 × ); total number of neoplastic cells counted (e.g., 500 or 1000 cells); regions of interest (e.g., hot spots or random spots); (4) instruments used for observation (e.g., visual count under the microscope only or visual count in combination with a digital microscope-assisted image analyzer); and (5) the rater's measurement experience. Second, only 1 rater measured the KIs and AIs; therefore, interrater variability was not elucidated. Nevertheless, the present study provides important information about sampling biases inherent to TMAs. Because measurement results can significantly differ when smaller samples are included in TMAs, interrater variability will be an issue even when a constant measurement method is employed by highly experienced pathologists. Further explorations will be necessary to identify a better sampling and counting strategy for biomarker studies that can minimize inter- and intrarater variability and precisely predict clinical outcomes.

In conclusion, our study suggests that KI and AI measurement results for TMA-based bladder urothelial carcinoma samples can be susceptible to systematic bias, and the lack of correlation between biomarkers cannot be avoided as it is with whole mount tissue preparations. Virtual TMAs in combination with Bland–Altman plot analysis can be useful for identifying a systematic bias before actually puncturing tissue blocks, and this will help establish a better sampling strategy for high-throughput TMA-based biomarker studies.

## Acknowledgments

The authors thank Junzo Kawaguchi, President of E-Path Co., Kanagawa, Japan, for providing useful advice on digital microscope-assisted morphometry.

## Supplementary Material

Supplemental Digital Content
