# Supplementary figures and images for: Comparison between whole mount tissue preparations and virtual tissue microarray samples for measuring Ki-67 and apoptosis indices in human bladder cancer: A cross-sectional study
Source: Medicine (Baltimore). 2016 Aug 7;95(31):e4500. doi: 10.1097/MD.0000000000004500 (PMC4979853; doi:10.1097/MD.0000000000004500)

## Slide 1
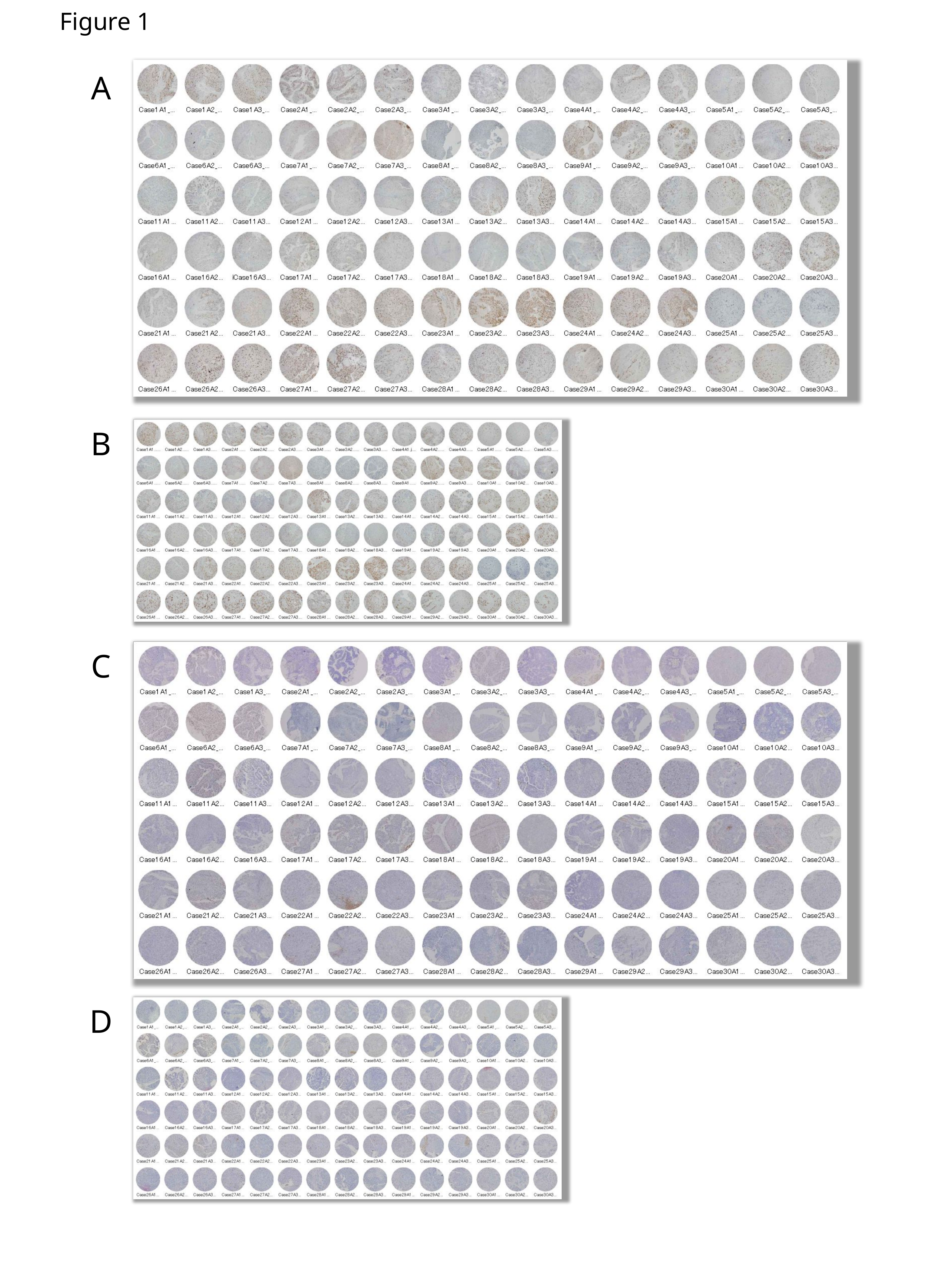

Figure 1
A
B
C
D

Supplement: Supplemental Digital Content [file medi-95-e4500-s001.pptx]
